# Placental Evidence of Intrauterine Stress: Mechanisms, Timing, and Links to Perinatal Brain Injury

**DOI:** 10.3390/jcm15114380

**Published:** 2026-06-05

**Authors:** Charlotte F. Kim, Chrystalle Katte Carreon

**Affiliations:** 1 Department of Pathology and Immunology, Baylor College of Medicine, Texas Children’s Hospital, 6621 Fannin St., MC-1195, Houston, TX 77030, USA; 2 Department of Pathology, Harvard Medical School, Boston Children’s Hospital, Boston, MA 02115, USA; katte.carreon@childrens.harvard.edu

**Keywords:** placental pathology, intrauterine stress, perinatal brain injury, hypoxic-ischemic encephalopathy, fetal vascular malperfusion, placental inflammation, umbilical cord abnormalities, placental reserve, nucleated red blood cells, meconium

## Abstract

Placental examination is frequently performed following adverse fetal or neonatal neurologic outcomes to elucidate the cause or timing of injury. In practice, however, the placenta more often serves as a biologic archive of fetal stress, adaptation, and reduced physiologic reserve rather than a definitive record of injury. This review synthesizes current research linking placental pathology to perinatal brain injury, with emphasis on biological mechanisms and temporal interpretation. We examine how placental findings inform assessments of intrauterine stress, impaired placental or fetal perfusion, inflammation, and diminished placental reserve. Surrogate markers of fetal stress, including nucleated red blood cells and intrauterine meconium passage, provide contextual evidence regarding the duration and severity of intrauterine compromise but do not establish causality. Finally, we review acute circulatory disruption, chronic or intermittent impairment of fetal blood flow, umbilical cord-related pathology, and immune-mediated placental disorders that increase vulnerability to neurologic injury.

## 1. Introduction

Placental examination is commonly performed following adverse fetal or neonatal neurologic outcomes, with the expectation that it will clarify the cause, mechanism, or timing of injury. In practice, however, the placenta serves less as a definitive record of discrete events and more as a biological archive of fetal stress, adaptation, and resilience. When interpreted within the appropriate clinical context, placental findings can yield important insights into intrauterine conditions, informing mechanistic understanding and timing, though they do not necessarily establish causation.

This interpretive complexity is particularly significant in the evaluation of perinatal brain injury, where multiple overlapping pathways, including altered perfusion, inflammation, and diminished placental reserve, converge during critical periods of neurodevelopment. Preterm birth further increases this vulnerability and is among the strongest and most consistent risk factors for neurologic injury. The immature brain is highly susceptible to hypoxic, inflammatory, and hemodynamic disturbances, with characteristic injury patterns such as periventricular leukomalacia reflecting this developmental sensitivity. Placental pathology is important in this context, as inflammatory and vascular lesions are disproportionately observed in preterm deliveries and may contribute both to the initiation of preterm birth and to the intrauterine environment in which injury occurs. Additionally, clinical interventions such as antenatal magnesium sulfate, which has been shown to reduce the risk of cerebral palsy in impending preterm delivery [1], highlight the importance of identifying fetuses at risk, even when such interventions do not alter the underlying placental pathology.

In clinical practice, placental findings are interpreted alongside other sources of information, including neuroimaging and laboratory data. Modalities such as cranial ultrasound and magnetic resonance imaging remain central to the diagnosis and characterization of perinatal brain injury, while biochemical markers may provide additional evidence of fetal stress or injury [2,3,4,5]. These complementary data sources enhance clinical interpretation but do not diminish the placenta’s distinct role as a record of intrauterine conditions.

Importantly, placental findings must also be interpreted within a broader differential framework. Genetic and congenital conditions, including those associated with consanguinity [6,7] may independently contribute to adverse neurologic outcomes, particularly when associated with central nervous system malformations. These factors can clinically overlap with placental-mediated injury and should be considered in a comprehensive evaluation, especially when placental findings appear disproportionate to clinical severity.

In this review, we synthesize current evidence linking placental pathology to fetal and neonatal brain injury, with particular emphasis on mechanistic plausibility, temporal interpretation, and the inherent limitations of causal inference. Rather than defining the clinical or diagnostic features of brain injury, the objective is to highlight the role of the often-overlooked placenta as a critical interpretive substrate, which may function as a causal contributor, a corollary marker, or an adaptive record of intrauterine stress.

## 2. Discussion

### 2.1. Scope and Interpretive Limits of Placental Examination

Although numerous placental lesions have been statistically associated with adverse neurologic outcomes [8,9,10,11], absolute causal relationships between specific placental lesions and neurologic injury are rarely demonstrable due to ethical limitations of prospective experimental trials of human pregnancy. Instead, placental pathology must be understood as contextual evidence within the broader clinical picture, helping to reconstruct the intrauterine environment in which neurologic injury may have occurred. When interpreted in aggregate and integrated with obstetric events and neonatal findings, placental pathology can provide meaningful insights into biologic plausibility and timing, even when it cannot establish singular causation.

### 2.2. Inferring Timing of Intrauterine Insult

#### 2.2.1. Nucleated Red Blood Cells

Some placental and fetal findings are best interpreted as indirect evidence of intrauterine conditions rather than as proximate causes of injury. Circulating nucleated red blood cells (NRBCs) fall squarely into this category. Rather than having a direct pathogenic effect, NRBCs represent a physiologic response to altered intrauterine homeostasis, most commonly hypoxia. Their value lies in their potential to inform *when* and *for how long* a fetus has been exposed to adverse conditions, while underscoring the inherent limitations of using surrogate markers to infer timing.

Beyond the first half of gestation, NRBCs are typically no longer identifiable in the fetal circulation [12]. Low-level detection near delivery, however, is not uncommon and may occur even in otherwise uncomplicated births.

NRBCs are immature erythrocytes (normoblasts) that still retain their nuclei. These cells are erythroid precursors that typically undergo nuclear extrusion within hematopoietic organs before entering the bloodstream as mature erythrocytes. Under stress conditions, this maturation sequence may be bypassed, allowing nucleated forms to appear in peripheral blood.

A range of intrauterine exposures may provoke NRBC release, including hypoxia, anemia, infection, and maternal diabetes [13,14,15,16]. Early responses are characterized by mobilization of pre-existing normoblasts, whereas sustained stimulation results in broader activation of erythropoiesis and the appearance of increasingly immature precursors. In rare circumstances of prolonged hypoxia compatible with fetal survival, primitive erythroblasts may circulate, reflecting profound and chronic hematopoietic activation. In these circumstances, erythropoiesis may occur in atypical visceral sites, including, but not limited to, the kidney and pancreas.

The mechanisms driving NRBC elevation differ by context. Hypoxia stimulates erythropoietin-dependent pathways [17], while infection likely acts through inflammatory signaling, with hypoxia often serving as a secondary contributor [13,18]. Maternal hyperglycemia may amplify these effects via increased fetal erythropoietin production [15,16]. These overlapping pathways underscore that NRBC elevation is not specific to a single insult.

Current evidence indicates that NRBCs may appear in circulation within hours of hypoxic exposure, challenging earlier assumptions that their presence requires days of augmented erythropoiesis [12,19,20]. As stress continues, NRBC counts rise, indicating both the severity and duration of the underlying condition. When hypoxia is chronic, elevated NRBCs may persist after birth and decline gradually over days [21,22,23,24]. Continued postnatal elevation should prompt consideration of additional etiologies.

NRBCs are reported either as relative counts per 100 leukocytes or as absolute concentrations.

In our experience, semiquantitative histologic estimates of NRBCs per 100 WBCs correlate well with automated counts from initial neonatal labs, provided the sample is drawn shortly after delivery. In uncomplicated deliveries, there is excellent agreement between automated absolute NRBC counts and automated counts expressed per 100 WBCs [25]. Absolute values are generally more informative when leukocyte counts are abnormal or rapidly changing. High early neonatal NRBC levels in conjunction with placental evidence of chronic stress have been associated with adverse neurologic outcomes; however, NRBCs themselves are not injurious and cannot identify the specific placental mechanism responsible.

Although NRBC burden and clearance patterns are widely used to infer intrauterine stress, reported associations with neurologic injury are inconsistent. Methodological differences, including threshold selection and study design, likely account for some inconsistency. Importantly, studies relying solely on neonatal laboratory data cannot distinguish timing or mechanism of injury without parallel placental correlation. NRBCs, therefore, provide context rather than causation and must be interpreted as part of an integrated clinicopathologic assessment.

#### 2.2.2. Meconium

Intrauterine meconium passage reflects a complex interplay between fetal maturation, particularly that of the enteric nervous system, and physiologic stress. Although meconium is more frequently observed with advancing gestational age and may occur in small amounts late in normal pregnancy, substantial experimental and clinical evidence support its release as a response to fetal compromise, particularly hypoxia. Meconium-stained amniotic fluid is consistently associated with adverse neonatal indicators, including acidemia, low Apgar scores, and increased need for intensive care [26,27], reinforcing its relevance as a marker of intrauterine stress.

Gestational age strongly influences the interpretation of meconium, as meconium passage during late gestation may reflect physiologic maturation of the fetal gastrointestinal tract [28]. Prior to the third trimester, meconium passage is uncommon even in severely compromised fetuses; therefore, its absence at earlier gestations does not exclude significant intrauterine stress. When present, meconium release appears most likely during an intermediate temporal window—events lasting hours to days—rather than during hyperacute catastrophes or prolonged, slowly progressive hypoxia, both of which may fail to elicit passage due to insufficient time or fetal adaptation.

Both acute and chronic hypoxic conditions have been implicated in triggering intrauterine meconium passage. Erythropoietin (EPO), which rises in response to sustained hypoxia and peaks approximately 48 h after insult onset, has been correlated with the presence of meconium in amniotic fluid in multiple studies [29,30,31]. These observations suggest an association with subacute or chronic hypoxia. In contrast, acute hypoxic events have been linked to elevated corticotropin-releasing factor (CRF), a hormone that stimulates colonic peristalsis; experimental models have demonstrated a strong relationship between CRF elevation and intrauterine meconium passage [32].

### 2.3. Meconium as a Pathologic Agent, Not Just a Marker

Once released, meconium interacts with placental tissues in a time-dependent manner. Contact with the amniotic epithelium initiates phagocytosis by macrophages within the membranes, chorionic plate, and umbilical cord. The depth of macrophage infiltration correlates with the interval between release and delivery, while the density of pigment reflects meconium burden [33].

Recently deposited meconium is typically superficial, removable from the amniotic surface, and clinically described as particulate, and sometimes referred to as “loose meconium.” With increasing duration, pigment becomes embedded in tissue, darkens, and cannot be mechanically removed. Importantly, gross green or brown discoloration is not specific to meconium and may result from blood breakdown products or inflammatory processes.

**Figure 1:**
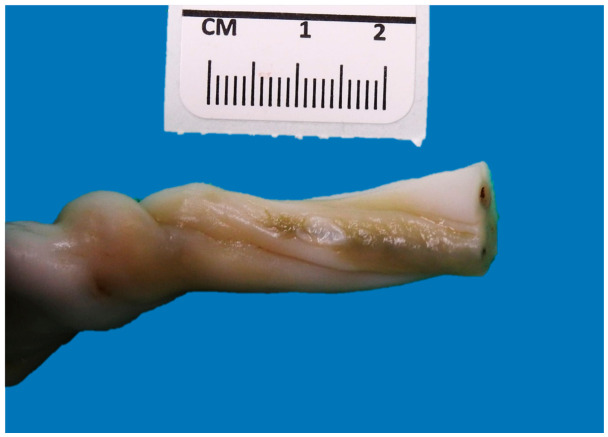
Prolonged intrauterine meconium exposure with umbilical cord ulceration. Gross photograph showing focal ulceration of the umbilical cord surface associated with sustained exposure to meconium. This lesion develops over hours to days and reflects prolonged intrauterine stress rather than an acute intrapartum event.

Meconium is not merely a byproduct of stress but can contribute directly to placental and fetal injury. Prolonged exposure, particularly within the umbilical cord, may result in meconium-associated vascular myonecrosis, a lesion linked to altered vascular tone [34,35,36,37,38] that is independently associated with adverse neonatal neurologic outcome [34,35,36,37,38]. Experimental studies assessing direct exposure of umbilical cords to meconium have shown that histologic evidence of vascular necrosis develops only after sustained contact, implying that meconium release typically predates delivery by many hours in these cases [39,40]. Clinically, this interpretation is reinforced by the observation that nearly half of placentas with meconium-associated myonecrosis are accompanied by thick meconium, a finding suggestive of prolonged exposure [41]. Gross examination often reveals deep green or green–brown discoloration of the membranes and umbilical cord and may show gross ulceration of the chorionic plate or umbilical cord vessels (Figure 1).

The macroscopic appearance of meconium varies with duration. Recently released meconium typically causes a superficial green discoloration of the amniotic membranes and chorionic plate, which may be patchy and easily removed from the amniotic surface. Clinically, this presentation is often described as “particulate” meconium, a feature that corresponds microscopically to amorphous orange–brown material adherent to the amniotic surface. With increasing duration, meconium becomes incorporated into tissue, cannot be mechanically removed, and evolves toward a darker green–brown or brown coloration. Importantly, these color changes are not specific to meconium, as similar hues can result from blood degradation products or from neutrophil myeloperoxidase activity in infectious chorioamnionitis.

Histologically, true meconium is characterized by fine, opaque, nonrefractile orange–brown pigment within macrophage cytoplasm. Surface meconium not yet phagocytosed appears amorphous and globular. Over time, partial enzymatic digestion produces a paler, vacuolated, or “bubbly” appearance. The burden of pigmented macrophages correlates with meconium concentration, whereas their depth of infiltration reflects the interval between meconium passage and delivery.

Clinically, meconium is often categorized by thickness. Although formal three-tiered grading systems exist [42], dichotomous classification of thin versus thick meconium is most commonly used. Meconium thickness generally reflects both the severity and duration of the inciting stress. Thick meconium shows a strong association with abnormal fetal heart rate tracings and low Apgar scores and is more frequently linked to adverse outcomes than thin meconium [43,44]. While amniotic fluid volume may modulate apparent density [44], thick meconium is widely regarded as a marker of non-acute fetal compromise.

In addition, meconium possesses bioactive properties capable of inducing a sterile inflammatory response [45,46,47]. Its bile acid components promote neutrophil chemotaxis and may produce histologic patterns that resemble those of infectious chorioamnionitis. In meconium-driven inflammation, however, the fetal inflammatory response often predominates over the maternal response, a distinction that requires careful clinicopathologic correlation.

Finally, caution is warranted in interpreting pigmented macrophages within placental tissues. Hemosiderin, bilirubin, and other endogenous pigments may mimic meconium, particularly in the absence of supporting clinical or gross findings. Because no special stain reliably distinguishes meconium from other pigments, accurate interpretation depends on integration of clinical history, gestational age, gross examination, and accompanying histologic changes.

### 2.4. Acute Disruption of Oxygen or Blood Delivery to the Placenta

Some causes of perinatal brain injury arise not from progressive placental dysfunction but from abrupt failures of oxygen delivery or blood volume maintenance. These events are characterized by sudden interruption of maternal or fetal circulation and are typically attributable to mechanical disruption or vascular compromise rather than to chronic placental disease.

Acute maternal perfusion failure is most often the consequence of catastrophic obstetric events such as uterine rupture, placental abruption, or maternal circulatory collapse. These events may rapidly curtail uteroplacental blood flow, leading to profound fetal hypoxia over a matter of minutes. While the placenta is generally regarded as having considerable functional reserve, clinical experience indicates that placental separation involving roughly 50% of the disc is usually fatal [48], but even smaller abruptions in our experience can lead to fetal injury [49,50,51] or even demise if other pathologies are present. Accordingly, placental and uterine pathology captures both the severity of ischemic injury and the interval between the insult and delivery or demise. In cases where placental separation progresses over time or recurs, pathologic examination commonly reveals lesions of differing ages.

**Figure 2:**
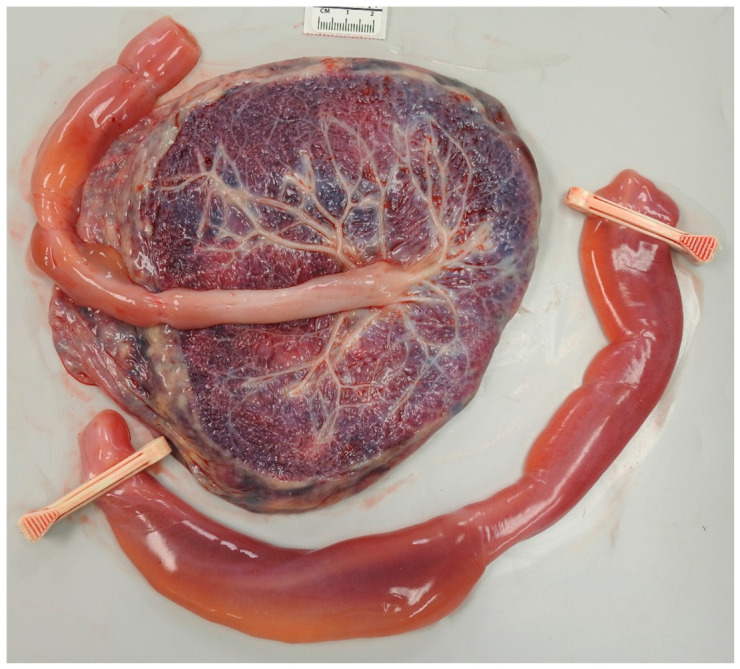
Massive fetal hemorrhage with acute depletion of fetal blood within placental vessels. Gross photograph showing pale chorionic plate vessels devoid of blood, reflecting acute fetal blood loss and circulatory collapse from massive fetal hemorrhage rather than chronic placental pathology.

Vasculature that lack the protective support of Wharton’s jelly, such as membranous vessels in velamentous cord insertion, is especially susceptible to rupture. Although complete transection of the umbilical cord is rare, even partial vascular disruption may lead to significant fetal hemorrhage and is associated with a high rate of perinatal mortality [52]. Accordingly, meticulous pathologic examination is critical for demonstrating antepartum vessel injury and distinguishing true pathologic findings from postdelivery artifact, particularly in cases with limited clinical context. By comparison, the placental manifestations of severe fetal anemia are generally more readily apparent. Because the volume of blood within the chorionic vessels and villous capillaries—and therefore their gross coloration—closely reflects the fetal hematocrit, marked anemia typically produces a strikingly pale placental parenchyma with chorionic plate vessels that appear devoid of fetal blood (Figure 2).

Fetal blood loss may also occur through leakage of blood from villous capillaries into the maternal circulation, producing fetomaternal hemorrhage. This process often lacks an identifiable traumatic trigger and may be episodic, resulting in cumulative anemia rather than sudden exsanguination. Placental findings in such cases are nonspecific, and confirmation requires detection of fetal erythrocytes in maternal blood using established laboratory assays. Test interpretation must account for conditions that alter fetal cell persistence in the maternal circulation.

Monochorionic twin gestations present an additional setting in which acute circulatory imbalance can occur. While chronic twin-to-twin transfusion is well recognized, sudden transfer of blood from one twin to the other may follow fetal demise of a cotwin or acute hemodynamic shifts across pre-existing anastomoses. The surviving twin may rapidly develop severe anemia and hypovolemia, with neurologic injury occurring over a very short interval. This mechanism, rather than embolic phenomena [53], best explains the high morbidity observed in these situations [51,53,54].

Traumatic injury, including iatrogenic vascular puncture or cord injury during invasive intrauterine procedures [51,53], represents another mechanism of acute fetal blood loss. The organization and composition of associated hematomas may provide limited temporal information but should be interpreted cautiously. Umbilical cord hematoma represents a specific manifestation of such vascular disruption, typically resulting from rupture of an umbilical vessel within the cord [55]. In addition to rapid fetal blood loss, accumulation of extravasated blood may compress adjacent vessels, leading to interruption of blood flow and sudden hypoxia, with resultant risk of neurologic injury in surviving neonates.

Severe fetal anemia may also arise in the absence of overt vascular disruption. Infectious, immune-mediated, and inherited hematologic disorders can impair red cell survival or production, leading to reduced oxygen-carrying capacity with neurologic consequences comparable to hemorrhagic causes. In these settings, placental findings are often supportive rather than diagnostic, and definitive attribution requires integration of clinical history, laboratory evaluation, and placental examination.

### 2.5. Persistent or Intermittent Impairment of Fetal Blood Flow

Fetal vascular malperfusion (FVM) describes a constellation of placental findings that arise from sustained or intermittently reduced blood flow within the fetal circulation. Rather than reflecting an acute terminal event, FVM captures the placental consequences of impaired perfusion over time and is now recognized as a frequent substrate for adverse perinatal neurologic outcomes. Although the terminology was standardized relatively recently, the lesions grouped under FVM represent long-recognized patterns of fetal circulatory compromise.

**Figure 3:**
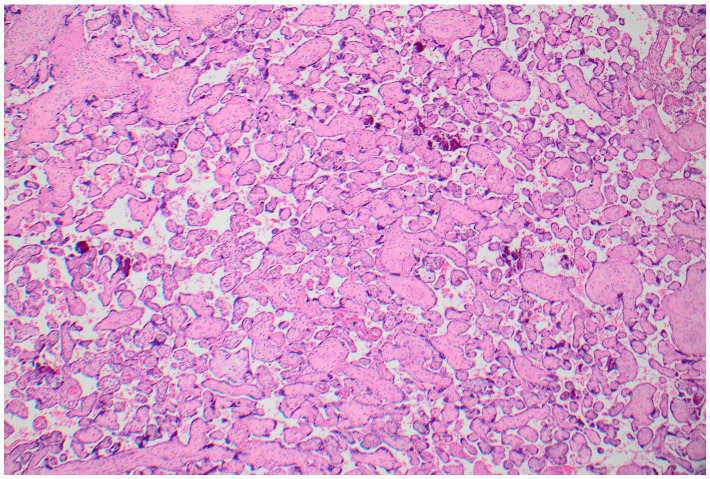
High grade fetal vascular malperfusion due to an abnormal umbilical cord insertion. Histologic section demonstrating sheets of avascular villi in the setting of fetal vascular malperfusion secondary to a velamentously inserting umbilical cord. The distribution of avascular villi indicate sustained or recurrent impairment of fetal blood flow, a histologic diagnosis associated with increased vulnerability to perinatal brain injury.

Histologically, FVM is characterized by thrombosis within large fetal vessels and downstream ischemic changes affecting the placental villi. These changes include loss of normal villous capillary networks and involutional degeneration of villous stromal structures, reflecting withdrawal of fetal perfusion from previously viable territories (Figure 3).

Clinically, placentas with FVM are often associated with decreased fetal movement, intrapartum fetal heart rate abnormalities, stillbirth, and increased risk of hypoxic–ischemic encephalopathy and/or cerebral palsy cases [56,57,58,59,60], and perinatal stroke [61].

While intrinsic fetal conditions such as congenital heart disease or inherited coagulation disorders may contribute in a subset of cases, the most common pathway leading to FVM involves mechanical or functional obstruction of blood flow at the level of the umbilical cord. Multiple cord-related abnormalities may act alone or in combination to limit fetal perfusion.

Doppler ultrasound provides complementary physiologic insight into fetal and placental hemodynamics. Abnormal umbilical artery Doppler indices, including elevated resistance, absent end-diastolic flow, or reversed flow, reflect increased placental vascular resistance and are strongly associated with fetal growth restriction and adverse neurologic outcomes [62,63]. Assessment of venous Doppler parameters may further identify impending fetal decompensation [64]. In addition, Doppler assessment of umbilical cord characteristics, including coiling and flow resistance patterns, may provide indirect evidence of cord-related compromise that contributes to impaired fetal perfusion [65]. In this context, Doppler findings serve as functional correlates of the impaired blood flow that, when sustained, may culminate in the histologic patterns of fetal vascular malperfusion observed at delivery.

### 2.6. Umbilical Cord Compression

The umbilical cord represents the fetus’s sole circulatory connection to the placenta, rendering fetal oxygen delivery uniquely vulnerable to obstruction. Most forms of cord-related compromise arise from mechanical compression, which may be transient, intermittent, or sustained. Compression can result from external forces such as cord entanglement, prolapse, or reduced amniotic fluid volume, as well as from intrinsic abnormalities of the cord itself.

All of these conditions have been associated with adverse outcomes, including fetal demise, neurologic injury, and abnormal developmental outcomes [50,53,66,67,68,69,70,71,72]. When obstruction is sudden and complete, fetal death is common; among survivors, significant neurologic damage is likely [50,51,67,70,73,74,75]. Similarly, prolonged partial obstruction can lead to serious consequences. Chronic cord conditions such as long cords, hypercoiling, constrictions, and velamentous insertion are disproportionately represented in placentas from infants with later neurologic impairment [49,67,74,75], suggesting that long-standing reductions in perfusion may be particularly injurious.

Labor frequently represents a period of increased vulnerability. Changes in fetal position, descent through the birth canal, or rupture of membranes may convert a previously compensated cord abnormality into a hemodynamically significant event. Because the umbilical vein is more distensible than the arteries, compression preferentially impairs venous return [70], exacerbating fetal hypovolemia and limiting oxygen delivery even as arterial outflow continues. Placental examination may identify a predisposing cord abnormality and, in such cases, may reveal dilation of umbilical or chorionic plate vessels and localized degenerative changes within Wharton’s jelly, ischemic necrosis of umbilical vessel walls (likely related to pressure-type necrosis), and occasionally fetal vasculitis in response to cellular injury.

Cord entanglement, true knots, and cord prolapse are often considered together because each represents a mechanical abnormality capable of partially or completely obstructing umbilical blood flow. The umbilical cord may become looped around virtually any fetal body part; however, involvement of the neck, commonly referred to as a nuchal cord, is by far the most frequent configuration. Entanglements have been documented early in gestation, including during the first trimester, and nuchal cords are commonly identified during routine obstetric ultrasonography [76], although some resolve spontaneously before delivery. The prevalence of nuchal cords increases with advancing gestational age and is observed in approximately one-third of term pregnancies [77]. While a single loop is most typical, multiple loops may occur, and rare cases of extensive looping have been reported [53,78].

Not all nuchal cords carry the same clinical significance. Loose, freely sliding loops typically have minimal hemodynamic impact, whereas tight entanglements are more likely to impair blood flow and are associated with less favorable outcomes [79]. The association between nuchal cords and fetal growth restriction suggests that some entanglements reflect prolonged intrauterine conditions rather than isolated intrapartum events [80]. Although many entanglements remain clinically silent, tightening may occur during labor, particularly after membrane rupture and fetal descent. Tight entanglements are associated with increased rates of low Apgar scores, perinatal complications, and fetal demise [67].

True knots of the umbilical cord constitute another form of potentially obstructive lesion and, like entanglements, may be either loose or tight. Loose knots are often incidental findings without clinical consequence. In contrast, tight knots may significantly restrict blood flow and are characterized pathologically by venous distension distal to the knot, placental congestion, and, in some cases, thrombosis of surface vessels. True knots are relatively uncommon and are encountered more frequently in the setting of long or excessively coiled cords [51,53,68]. They have been associated with intrapartum fetal heart rate abnormalities, fetal distress, hypoxia, neurologic injury, and increased perinatal morbidity [53,81].

Cord prolapse represents a distinct, typically acute, mechanism of umbilical cord compression. In this scenario, the cord descends ahead of the presenting fetal part during labor or delivery and becomes compressed between the fetus and the maternal pelvis. Although cord prolapse is infrequent, occurring in less than one percent of deliveries, it carries a substantial risk of perinatal death and neurologic injury [53]. Risk factors include abnormal fetal presentation, low birth weight, obstetric manipulation, polyhydramnios, abruption, placenta previa, and a long umbilical cord [82]. Predisposing factors include abnormal presentation, low birth weight, obstetric manipulation, excess amniotic fluid, placental abnormalities, and excessive cord length. Pathologic examination may demonstrate acute vascular congestion and, in some cases, localized evidence of compression injury.

Anatomic variations in cord insertion further influence vulnerability to compromised blood flow. Marginal, defined as cord insertion ≤ 1.0 cm from the disc edge [83], and velamentous insertions place fetal vessels at greater risk by reducing their mechanical support and protective buffering. In velamentous insertion, fetal vessels traverse unsupported membranes and are therefore particularly susceptible to compression, thrombosis, or rupture, especially after membrane rupture or during labor. Although hemorrhage from ruptured velamentous vessels occurs in only about 1 in 50 cases, mortality from subsequent fetal hemorrhage is high [84]. Marginal cords are more commonly associated with NICU admission [85], FVM [86], and severe HIE [87].

Cord caliber represents another important determinant of vulnerability. Thin umbilical cords, characterized by reduced Wharton’s jelly, are less resistant to compression and are often associated with impaired fetal growth and placental vascular pathology. Cord diameter correlates with overall fetal and placental health, and a reduced diameter may limit perfusion within the fetal circulation. These features are encountered with increased frequency in placentas demonstrating FVM [73].

Careful gross examination of the placenta is essential in identifying insertion abnormalities, reduced cord caliber, and vessel disruption, particularly in cases of unexplained fetal compromise or neurologic injury.

#### 2.6.1. Umbilical Cord Configuration

The structural configuration of the umbilical cord contributes substantially to its ability to withstand mechanical stress. Features such as coiling, diameter, and length are established early in gestation and reflect complex interactions among fetal movement, hemodynamic forces, and developmental factors. Departure from typical patterns may increase susceptibility to vascular compromise.

Normally, the number of coils in the cord is about 2 coils/10 cm, which is referred to as the coiling index [70]. The physiologic mechanisms underpinning cord coiling are not fully understood but are thought to involve hemodynamic factors, anatomical factors such as the volume of amniotic fluid, genetic influences, and fetal activity in utero [88]. Excessive coiling can compress umbilical vascular lumina and increase resistance to flow, while insufficient coiling diminishes elasticity and resilience. Certain abnormal coiling patterns appear particularly prone to impaired perfusion and are overrepresented in placentas showing features of FVM [70,71,78,88,89,90]. Focal constrictions of the cord, most commonly near the fetal insertion, may further exacerbate flow restriction and are often accompanied by upstream congestion or thrombosis. Numerous studies have documented the adverse outcomes associated with umbilical cord stricture, including fetal demise, growth restriction, and intolerance to labor [53,70,73,74,91].

Cord length also modulates risk. Umbilical cords progressively lengthen throughout gestation until term, when the umbilical cord at term averages 53–60 cm [92,93]. Excessively long cords, generally defined as >70 cm at term, are associated with entanglement, knot formation, hypercoiling, and increased intraluminal resistance, all of which may interfere with effective perfusion. These cords are frequently accompanied by placental markers of chronic hypoxia [68]. Excessive cord length has also been associated with increased morbidity, including brain imaging abnormalities [94] and poor long-term neurodevelopmental outcomes [68]. Although no universal definition exists, term cords measuring <40–45 cm are generally considered short, with excessively short cords defined as <25 cm [95]. Both extremes of cord length have been associated with increased perinatal morbidity, neurologic injury, and mortality [96,97,98].

#### 2.6.2. Fetal Thrombosis and Downstream Ischemia

Reduced fetal blood flow creates conditions favorable to vascular stasis, which may progress to thrombosis within large fetal vessels of the chorionic plate or stem villi. Such thrombi impair perfusion to downstream villous territories, initiating a cascade of ischemic and involutional changes.

As perfusion ceases, stem villi undergo sclerosis and collapse, often accompanied by extravasation and fragmentation of fetal red blood cells. In terminal villi, similar processes produce stromal–vascular fragmentation, representing an intermediate stage in the evolution of avascular villi. The extent, distribution, and maturity of these lesions reflect both the severity and duration of flow impairment and may vary depending on whether obstruction is localized or widespread.

Although mechanical obstruction of the umbilical circulation is the most frequent precipitating factor, thrombosis may also occur in association with prothrombotic tendencies, metabolic disease, cardiac dysfunction, or inflammatory states that promote stasis. Regardless of etiology, fetal vascular thrombosis has been repeatedly associated with adverse neurologic outcomes and remains a central feature of clinically significant FVM [58,99]. Placental thrombi have been linked to thrombotic lesions in the fetal brain and are associated with adverse outcomes, including stroke [50,68,100,101].

### 2.7. Inflammation-Mediated Pathways to Brain Injury

Inflammation within the intrauterine environment represents an important non-mechanical pathway linking placental pathology to fetal brain injury. Exposure of the fetus to inflammatory mediators—whether arising from infection or sterile triggers—can initiate systemic and neurologic effects that independently contribute to adverse neurodevelopmental outcomes or exacerbate coexisting hypoxic or perfusion-related injuries.

Ascending infection of the amniotic cavity provides the most extensively studied inflammatory model. In this setting, microorganisms typically reach the uterine cavity through the cervix, often in the presence of intact membranes, reflecting failure of host defensive barriers rather than membrane rupture [102]. The earliest inflammatory response is maternal in origin and is confined to maternal tissues of the fetal membranes. As microbial burden increases, inflammatory activity may extend beyond this compartment, allowing bacterial products to enter the amniotic fluid and exposing the fetus to potent inflammatory stimuli.

Once fetal exposure occurs, a distinct fetal inflammatory response may develop. This response is defined not simply by the presence of inflammatory cells, but by their anatomic origin. Maternal inflammation arises from the intervillous space as well as maternal vessels on the extraplacental membranes and basal plate. In contrast, fetal inflammation is characterized by the migration of leukocytes from fetal vessels within the chorionic plate and umbilical cord. Involvement of fetal vessels reflects systemic fetal activation and may be accompanied by endothelial injury and, in some cases, thrombosis within the fetal circulation. These changes generally evolve over days rather than minutes, underscoring the importance of duration in determining injury risk.

It is important to recognize that infection is not required for a fetal inflammatory pattern to emerge. Certain noninfectious exposures, most notably meconium, can provoke a sterile inflammatory response that closely mimics infection-related vasculitis. Experimental and clinical observations suggest that this response is mediated by cytokine-driven chemotaxis [47] rather than microbial invasion. As a result, histologic patterns resembling infectious inflammation may be encountered in the absence of identifiable pathogens, and inflammatory and meconium-related processes may coexist within the same placenta.

From a neurologic perspective, fetal inflammation exerts its effects primarily through cytokine action rather than direct tissue invasion. Exposure to inflammatory mediators can occur without fetal infection and is sufficient to trigger widespread physiologic consequences. In the developing brain, cytokines may interfere with normal cellular maturation, alter vascular tone, and disrupt the integrity of the blood–brain barrier [49,103,104]. These effects increase vulnerability to ischemic injury and may result in direct cellular toxicity, particularly in the immature central nervous system.

When infection progresses to directly involve the fetus, additional mechanisms compound the risk of injury. Pulmonary involvement is common, reflecting aspiration of infected amniotic fluid, and impaired gas exchange may intensify hypoxia. Systemic infection may further compromise cerebral perfusion through hypotension and circulatory instability. Not surprisingly, severe fetal inflammatory responses have been associated with white matter injury, periventricular leukomalacia, and cerebral palsy [49,72,99,105].

Importantly, fetal exposure to inflammatory stimuli does not equate to inevitable brain injury. Multiple interacting variables, including gestational age, duration of exposure, intensity of the inflammatory response, and the presence of concurrent hypoxic or vascular insults, determine neurodevelopmental outcome. Accordingly, inflammatory placental findings must be interpreted within a broader clinicopathologic framework to distinguish causal mechanisms from coincident or incidental associations.

### 2.8. Chronic Reduction in Placental Reserve

A subset of placental disorders contributes to fetal brain injury not by producing a discrete acute insult, but by progressively limiting placental functional capacity. These conditions diminish the placenta’s ability to buffer physiologic stress, thereby increasing fetal vulnerability to superimposed hypoxic, hemodynamic, or inflammatory exposures. In some settings, they may also function as primary drivers of injury in the absence of an acute precipitating event.

#### 2.8.1. Maternal Vascular Pathology as a Modifier of Risk

Maternal vascular pathology originates from impaired remodeling and function of the uterine spiral arterioles, resulting in chronically reduced maternal blood delivery to the intervillous space. These disorders are typically persistent rather than episodic and are prone to recur in subsequent pregnancies when the underlying maternal condition remains uncorrected. The shared pathophysiologic feature is compromised uteroplacental perfusion, which restricts placental growth, alters villous development, and produces a recognizable pattern of ischemic change.

Conditions commonly associated with this pattern include chronic hypertension, pregestational diabetes, hypertensive disorder of pregnancy such as pre-clampsia or HELLP, and systemic autoimmune disorders. Less frequently, structurally abnormal uteri with intrinsically limited vascular supply may contribute. The role of inherited thrombophilias remains controversial, with inconsistent support across studies.

In significantly affected pregnancies, the placenta is often disproportionately small and demonstrates features of chronic villous stress [106], including distal villous hypoplasia, aggregated terminal villi, villous hypermaturation, increased syncytial knots, and intervillous cytotrophoblast/fibrinoid islands, as well as decidual arteriopathy. These include abnormalities in villous architecture consistent with reduced perfusion and altered maternal–fetal exchange. A defining feature is pathology of the decidual spiral arterioles. Mild disease is characterized by incomplete physiologic transformation of these vessels, with persistence of smooth muscle and increased vascular resistance. More advanced lesions show structural breakdown of the vessel wall replaced by dense, proteinaceous material; inflammatory lipid-laden macrophages may be present in the most severe forms. These abnormal vessels are predisposed to thrombosis and loss of integrity, creating a substrate for placental infarction and abruption. Chronic reduction in maternal blood flow renders the fetal vascular tree underdeveloped, with reduced branching and narrowed villous profiles and capillaries—a pattern reflecting long-standing flow limitation rather than acute injury.

Although many infants born from such pregnancies demonstrate no overt neurologic impairment—likely reflecting adaptive cerebroprotective responses—risk is not uniform. Infants who are small for gestational age, growth-restricted, or of very low birth weight appear particularly vulnerable [107,108,109]. The likelihood of adverse neurologic outcome correlates with the severity and extent of placental pathology and inversely with birth weight [108]. Importantly, maternal vascular malperfusion is not infrequently identified in placentas from infants with hypoxic–ischemic encephalopathy [59,72], supporting the concept that reduced placental reserve may lower the threshold for injury when additional stressors occur.

#### 2.8.2. Immune-Mediated Placental Disorders

A subset of chronic placental disorders appears to arise from dysregulated maternal immune interactions with the fetal–placental unit. These conditions are not associated with identifiable infection, yet they produce sustained inflammatory changes and/or alterations in the intervillous space that compromise placental exchange capacity and reduce tolerance to physiologic stress. Their contribution to fetal injury is best understood through the combined effects of structural disruption and immune-mediated signaling rather than through a single pathogenic mechanism. Hormonal mediators such as human chorionic gonadotropin (hCG) may also modulate immune and vascular interactions at the maternal–fetal interface [110,111], although their role in specific patterns of placental injury remains incompletely defined.

Villitis of unknown etiology (VUE) is a chronic inflammatory process characterized by the presence of maternal immune cells, most notably T lymphocytes and macrophages, within the villous stroma. Although its precise cause remains unresolved, converging clinical and molecular evidence supports an immune rejection-like process directed against fetal tissues [112,113]. In most cases, inflammation preferentially involves terminal villi, which constitute the principal sites of maternal–fetal exchange. In more extensive disease, inflammation extends into larger villous structures, where it may damage the fetal vascular endothelium.

**Figure 4:**
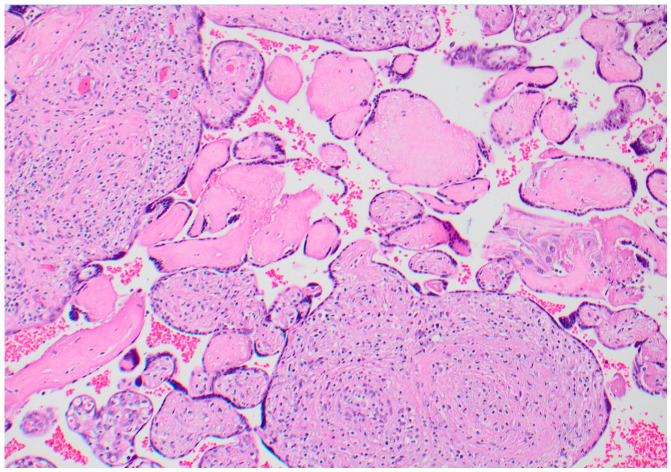
Chronic villitis of unknown etiology with downstream vascular obliteration. Histologic section showing high grade chronic villitis of unknown etiology with obliterative fetal arteriopathy and downstream avascular villi. This pattern reflects immune-mediated injury to the fetal-placental circulation, contributing to a chronic reduction in placental reserve and heightened susceptibility to neurologic injury.

When inflammatory injury within larger villi becomes severe, progressive narrowing or obliteration of fetal vessels may occur, interrupting blood flow to dependent villous territories. The downstream consequences of this process include degeneration of villous stromal and vascular elements and the eventual appearance of avascular villi (Figure 4).

This pattern overlaps mechanistically, though not etiologically, with fetal vascular malperfusion.

In addition to local structural injury, VUE is associated with systemic immune activation. Studies demonstrating differential chemokine expression in maternal and fetal compartments support the view that villitis represents a systemic immunologic milieu rather than a lesion confined to placental tissue [114,115]. The fetal inflammatory milieu associated with VUE differs from that seen in ascending infection, suggesting separate pathways of immune activation. These circulating mediators may exert neurotoxic effects or amplify vulnerability to concurrent hypoxic or hemodynamic stress.

Clinically, extensive or “high-grade” VUE, particularly when accompanied by vascular obliteration, is associated with fetal growth restriction, fetal demise, hypoxic–ischemic encephalopathy, and long-term neurologic impairment [11,49,72,116,117]. Disease burden, rather than mere presence, appears to be a key determinant of outcome.

Together, these observations underscore how chronic immune-mediated injury can compromise placental exchange capacity through both inflammatory and vascular pathways. Related placental disorders that similarly limit maternal–fetal perfusion—though through predominantly acellular rather than cellular mechanisms—are illustrated by maternal floor infarction and massive perivillous fibrin deposition. Maternal floor infarction (MFI) and massive perivillous fibrin deposition (MPFD) represent closely related forms of severe placental injury characterized by excessive accumulation of acellular material within the intervillous space. Although described separately based on distribution, both conditions share common structural and clinical features and are widely considered part of a disease spectrum.

In MFI, dense deposits line the maternal surface of the placenta, forming a rigid barrier between maternal blood and underlying villi. In MPFD, similar material extends throughout the placental parenchyma, entrapping villi across broad regions. Despite conventional terminology, these deposits are not composed solely of fibrin. Rather, they include protein-rich extracellular matrix derived in part from trophoblastic sources, likely reflecting altered placental metabolism in response to immune or hypoxic stress.

Functionally, these deposits restrict maternal blood flow to affected villi and may also impair perfusion to nearby regions that appear histologically uninvolved. Grossly, affected placental areas are firm and pale, in contrast to the normal spongy parenchyma. Microscopically, villi are encased within dense, eosinophilic material that limits exchange and promotes ischemic injury.

Both MFI and MPFD are strongly associated with adverse pregnancy outcomes, including recurrent pregnancy loss, prematurity, fetal growth restriction, stillbirth, and impaired neurodevelopment among survivors [118,119,120]. High recurrence rates in subsequent pregnancies and reports of response to immunomodulatory therapy support—but do not conclusively establish—a maternal immune contribution to pathogenesis [121].

### 2.9. Vascular Lesions

Placental chorioangioma is a benign vascular tumor of fetal origin. If large, this lesion can act as a low-resistance shunt in the placenta, overloading the fetal heart and potentially leading to high-output heart failure, anemia, or hydrops. These hemodynamic changes may reduce oxygen delivery to the fetus and increase the risk of neurologic injury, especially if other placental lesions are present. In addition, chorioangiomas have been associated with thromboembolic phenomena, with reported cases linking these lesions to downstream vascular events such as perinatal stroke [122].

### 2.10. Integrative Interpretation: Putting the Pieces Together

Placental lesions associated with neonatal brain injury do not operate through a single pathway. Instead, they act through overlapping mechanisms that limit oxygen delivery, disrupt fetal circulation, or expose the developing brain to inflammatory mediators. Some lesions produce abrupt physiologic collapse, while others exert their effects more subtly by eroding placental reserve over time.

Markers of fetal stress, such as elevated circulating nucleated red blood cells or the presence and distribution of meconium-laden macrophages, provide biologic evidence of intrauterine adversity but do not, in isolation, define the mechanism or timing. Their greatest interpretive value lies in contextual integration with placental structure, clinical history, and neonatal course.

Placental examination, therefore, plays a critical role in assessing the risk of neonatal neurologic injury—not as a tool for assigning singular causality, but as a means of reconstructing the intrauterine environment in which injury occurred. Recognition of both acute insults and chronic reserve-limiting conditions allows for a more accurate evaluation of vulnerability, timing, and biologic plausibility.

Many placental disorders, particularly those related to maternal vascular pathology or immune dysregulation, demonstrate recurrence in subsequent pregnancies. Recognition of these patterns extends the interpretive value of placental examination beyond a single gestation, allowing prior findings to inform risk stratification in future pregnancies. When integrated with clinical history, placental pathology can support more nuanced counseling regarding recurrence risk, guide surveillance strategies, and frame expectations for prevention. In this way, the placenta serves not only as a retrospective record of intrauterine conditions but also as a tool that informs ongoing clinical care.

## Data Availability

No new data were created or analyzed in this study. Data sharing is not applicable to this article.
